# Exploring sense of coherence (SOC) in relation to working conditions for employees with hearing loss

**DOI:** 10.1177/10519815251349523

**Published:** 2025-06-20

**Authors:** Sara Båsjö, Johanna Gustafsson, Sarah Granberg

**Affiliations:** 1 School of Health Sciences, Örebro University, Örebro, Sweden; 2 Faculty of Medicine and Health, Örebro University, Örebro, Sweden

**Keywords:** hearing loss, workplace, salutogenesis, sense of coherence, working conditions, occupational health

## Abstract

**Background:**

People with hearing loss (HL) report several challenging situations in working life (WL). HL can negatively affect, e.g., the ability to communicate, creating barriers and difficulties in WL. However, there is a lack of studies that investigates salutary aspects in relation to working conditions for the target group. Sense of Coherence (SOC) is a salutary concept widely used when investigating aspects in WL but is less explored in relation to HL.

**Objective:**

To explore the concept of Sense of Coherence (SOC) in relation to working conditions for employees with HL.

**Methods:**

An observational study with a cross-sectional design including people with HL in working age using hearing aids (HAs) or cochlear implants (CIs). Comparisons were made between participants “in work” and participants on “HL-related sick leave”.

**Results:**

The “in work” group reported a higher SOC compared to the “HL-related sick leave” group. The analysis showed significant differences between the two groups in all three dimensions of SOC with the “in work” group being better off in almost all investigated variables.

**Conclusion:**

SOC is a valuable concept for investigating working conditions for employees with HL. The results indicate that to increase the possibility of salutary working conditions, it is necessary to focus on consequences of HL, such as the need for recovery, autonomy and support. Addressing these aspects increases employees’ comprehension of and ability to manage the work situation and their sense of meaningfulness, which may act as a buffer against work-related stress and reduce the risk of sick leave.

## Introduction

Working life (WL) is directly related to the economic, physical, mental, and social well-being of individuals,^1^ which highlights the need to promote healthy WL. It has also been shown that WL has a positive effect on the individual's psychosocial needs and social status.^2^ In addition to individual well-being, productive employment with decent working conditions for women and men, including people with disabilities, is important for economic growth.^3^ While many people with disabilities want to participate in WL, research demonstrates a lower employment rate^1^ for this group. This is also the case for people with hearing loss (HL),^4^ as HL can negatively affect the ability to communicate, creating barriers and difficulties in WL.^5,6^ In Sweden, about 1.5 million adults live with a HL. Of these, almost half are of working age and they constitute approximately 12% of the workforce.^7^

HL is a disability with auditory and non-auditory consequences. The former relate to hearing and audibility, while the latter relate to psychosocial implications and everyday aspects of living with reduced hearing. An example of an auditory consequence is being unable to hear effectively in office meetings, while the non-auditory consequence related to this situation could be anxiety about the job being in jeopardy or missing information related to one's responsibilities at work. Additional non-auditory consequences of HL could be related to depression, increased isolation, irritability, and exhaustion.^8^ In WL research in relation to HL, there is a clear connection between the auditory and non-auditory consequences of HL. A lack of accommodated working conditions for employees with HL may increase the risk of work-related health problems^5^ such as fatigue,^6,9^ sleep problems, tiredness and concentration problems.^10,11^ Due to demanding oral communication situations at work, it has been suggested that people with HL experience a double workload, which can be described as including both the works tasks themselves and the extra effort needed to compensate for the HL during the working day. This compensation includes, for example, increased concentration in different interactions and the use of coping strategies to manage the situation and the consequences of fatigue.^11,12^ Such work-related health problems could increase the risk of sick leave^13^ and disconnection from work.^5^ Indeed, research demonstrates that people with HL are more often on sick leave and to a higher extent are dependent on unemployment and sickness benefits.^4,10,14^ These conditions make people with HL a vulnerable group in WL.

The auditory consequence of not being able to hear effectively at work can be managed with hearing aids (HAs) and/or assistive listening devices (ALDs). However, non-auditory consequences at work relate to different aspects of WL, such as working conditions, and hence need to be addressed differently.

### Working conditions

The term “working conditions” encompasses both the organizational and the social work environment, with the former including such things as management, communication, demands, requirements, resources, responsibilities, task assignment and participation, and the latter including social interaction, social support and collaboration with colleagues and managers.^15^

#### Organizational working conditions and HL

For employees with HL, Svinndal et al.^5^ highlighted several important organizational factors related to working conditions, such as flexibility in relation to working hours and work accommodations, and high control and low demands in relation to spoken communication.

Several other barriers related to organizational working conditions for employees with HL have been reported. These concern lack of knowledge about the consequences of HL (employers and people with HL)^5,16^ low control over the work situation compared to people with normal hearing,^17^ poor sound environment,^16,18^ poor accommodation^5,16,18^ and malfunctioning of ALDs.^16,19^ Svinndal et al.^5^ concluded that there is a need for functional support at the workplace to help people with HL remain active in WL.

#### Social working conditions in relation to HL

With regard to psychosocial working conditions, coping strategies to increase opportunities for interaction with co-workers, support from managers and co-workers in relation to structured oral communication and meetings, the overall workplace culture, and attitudes towards HL have been highlighted as important for employees with HL.^5,6,16^ In addition, access to service providers such as audiologists, psychotherapists, and psychologists appears to be important, as early information about the existence of service providers and further access to a variety of them resulted in less strain for the employee.^5^

However, an inaccessible psychosocial work environment has been reported in relation to reduced social interaction,^6,16,18^ which resulted in a sense of exclusion during social activities at work and withdrawal from these activities.^6^

Consequently, in order to increase knowledge about aspects important for healthy working conditions for employees with HL, both organizational and psychosocial factors that contribute to such working conditions need to be investigated.

### Salutary working conditions

In recent years, salutary aspects of WL for employees with HL have been highlighted as important.^20,21^ The salutogenic work perspective focuses on factors that contribute to the creation of health-promoting working conditions^22^ and thus salutary WL. Salutogenesis can be viewed as an overarching umbrella term encompassing several different theories. Common to these theories is a focus on human resources, abilities, and skills rather than inabilities and shortcomings. One of the most common salutogenetic theories and key concepts is “Sense of Coherence” (SOC) as described by Antonovsky.^23^ SOC can be described as a life perspective, i.e., the extent to which the person views life as coherent, structured and meaningful. In the context of work, SOC is described as the employee's perception of the comprehensibility, manageability, and meaningfulness of the work situation.^22^

*Comprehensibility* (the cognitive dimension) focuses on whether the employee perceives the work situation as structured, consistent, and clear. *Manageability* (the behavioural dimension) concerns internal and external resources, and whether and how the employee uses them to cope with the demands of the work situation. *Meaningfulness* (the motivational dimension) focuses on and includes the extent to which the employee perceives it as worthwhile to invest energy in becoming involved and committed to the work situation.^22,23^

Previous studies on SOC in relation to working conditions imply that employees with higher SOC experience a greater degree of social support, better self-reported health, higher work engagement^24^ and lower levels of stress^25^ than employees with lower SOC. People who are established in the labour market report a higher SOC than people not active in the labour market.^24^ Furthermore, people who are working exhibit a significantly higher SOC than people on sick leave.^26^ Although SOC has been investigated in several WL studies, no studies have been found that relate SOC to the working conditions of employees with HL.

Consequently, given that health-promoting working conditions are important for sustainable WL, and that employees with HL report negative experiences in relation to organizational and social working conditions, the aim of the study was to explore the concept of SOC, in relation to working conditions for employees with HL with the specific research question:

“From a SOC perspective, how do the working conditions of employees with HL who do and do not have periods of hearing-related sick leave differ?

## Method

### Study design

The study was a cross-sectional survey.

### Participants

The sample consisted of people of working age (18–67 years) with HL who use HAs or CIs. They were either in active paid employment (full-time or part-time) or on HL-related sick leave from paid employment, currently or during one or more periods in the last 12 months (HL-related sick leave). The participants with no periods of sick leave are hereafter categorized as the “in work” group, and those with periods of sick leave as the “HL-related sick leave” group.

### Materials

The survey consisted of two parts. The first was *Participant Characteristics,* and the second was *Work Characteristics,* where items addressed *Sense of Coherence.* Both parts contained different areas, each of which consisted of either single questions or indices. These questions/indices originated mainly from validated questionnaires and previous surveys on health and WL. The survey has also been used in a previous study investigating health factors for a sustainable work situation for the target group.^21^

#### Participant characteristics

The first area in this part focused on demographic aspects and included questions on gender, age, educational level, and economic burden (difficulty meeting regular expenses and difficulty dealing with an unexpected expense), all of which were retrieved from the National Public Health Survey.^27^ The latter question was of interest because previous studies have shown a positive connection between health and financial situation.^28^ To identify periods of “HL-related sick leave” during the last year, a question developed by the authors was formulated as follows: *During the last 12 months, have you been on sick leave due to a health problem that you believe originates from your HL*?

The second area dealt with hearing-related information. Four questions, developed by the authors, focused on HAs or CIs. The questions covered the number of HAs or CIs used, how often the participants use HAs/CIs, and whether HAs/CIs have had a positive impact on their quality of life (QoL).

The third area focused on information about WL. The questions about the participants’ current occupation and employment situation were retrieved from the National Public Health Survey.^27^ Occupational changes were assessed with two different questions: “change of occupation or workplace due to hearing-related problems” and how “change of occupation affected their possibility to stay in WL”. Both questions were developed by the authors.

#### Sense of coherence (SOC)

This part of the questionnaire focused on work characteristics, which divided into three areas based on the operationalization of the SOC. These areas dealt with the SOC dimensions of comprehensibility, manageability, and meaningfulness. The categorization of the different questions and indices within each of these areas was based on how the three dimensions are described in the literature.^23^

##### Comprehensibility

Comprehensibility was measured with the subscale *Process of Change* (change done through open dialogue, based on employees’ needs and comprehensive information about the process of change) from the questionnaire “Work Experience Measurement Scale” (WEMS).^29^ This subscale is an index and includes sub-questions related to the understanding of organizational changes and processes of change at work, and measures the extent to which the employee experiences the work situation as structured, clear, and consistent. The sub-questions are derived from research on SOC by Antonovsky and research about work psychology with a focus on regenerative work.^29^ Regenerative work includes sustainable and promotive work systems and organizations that may influence the employees’ experience that his or her resources are supported.^30^

##### Manageability

The area on manageability focuses on the extent to which employees experience that they have useful resources that enable them to cope with the demands of the work situation in terms of autonomy, time experience, skills development, and hearing-related accommodations as a facilitative factor.

The area included five indices and five single questions. Two indices concerned *autonomy,* (when, what and how to perform work) and *time experience* (having enough time to perform and finish work tasks).^29^ Further, two indices measured *skill development* (self-development, participation in training, and the employees’ competence being taken advantage of) (Labour Force Survey (LFS)),^31^ and *self-efficacy* (confidence in one's own abilities).^32^ Research has demonstrated that self-efficacy is related to SOC^33^ and can further be related to all dimensions depending on how the questions are formulated. In the current study, the questions are formulated to investigate how well the person can handle different situations. Consequently, the self-efficacy index was categorized within manageability.

The fifth index, *work accommodation* (developed by the authors and used in a previous study^21^), and two single questions, *use of assistive listening devices (ALDs) at work* and their *perceived usefulness*, which are based on the work of Bjarnason,^34^ describe the participants’ *hearing-related accommodations at work as a facilitative factor*. The index included questions about whether accommodations were provided at work in respect to the HL, and if the accommodations were relevant and sufficient in relation to the HL.

Furthermore, three single questions about *rest and recovery*, *balance between WL and leisure time* and *energy-demanding tasks outside of work*^31^ were also categorized within this area of SOC.

##### Meaningfulness

Meaningfulness focuses on factors that contribute to the employee's experience of engagement and the value of involvement and commitment in the work situation.

The area contained four indices: *internal work experience* (meaningfulness and challenges at work, enjoyment of work and sufficient variety in tasks) and *supportive working conditions* (support from colleagues in terms of feedback and practical help, routines, and a good atmosphere) from WEMS.^29^ Two indices were also used concerning *leadership* (support and appreciation from the manager, responsiveness to employees’ views, and conflict management) and satisfaction with *salary* from LFS.^31^

### Procedure

Data collection was conducted at two different time points.

**Figure 1.**
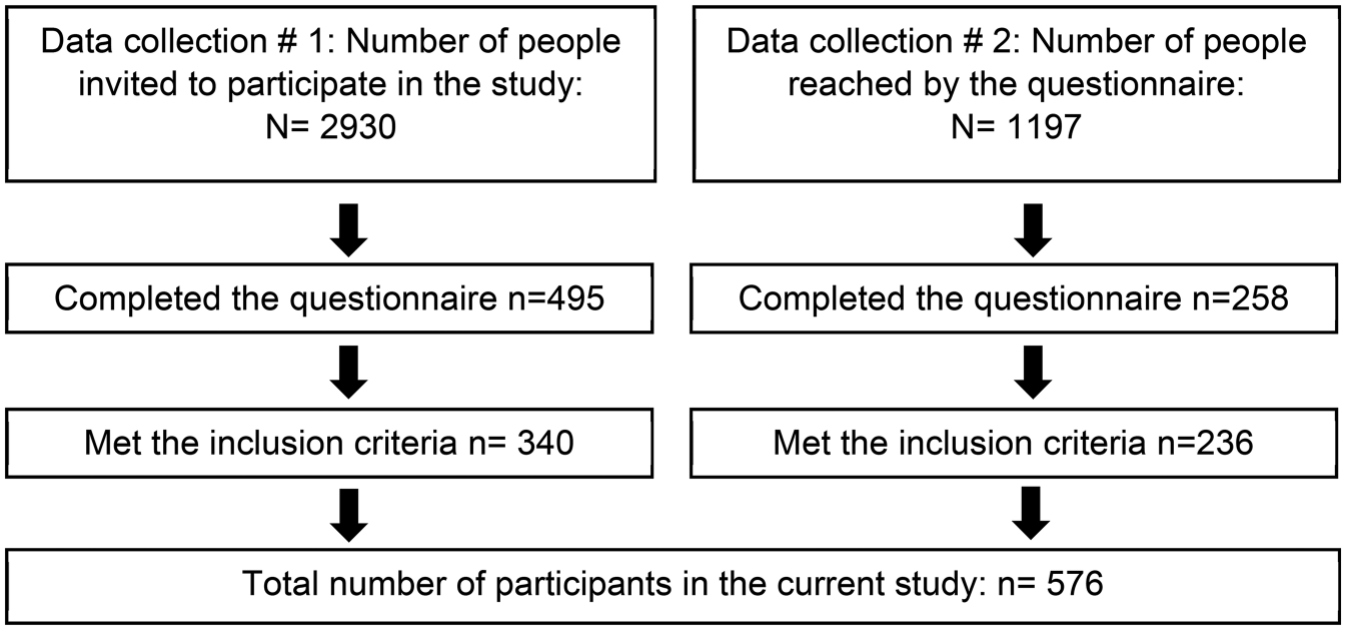
Flowchart of participant inclusion.

In the first data collection, adults of working age who were registered at the audiological clinic in Örebro County, Sweden, were invited to participate in the study. In total, 2930 people received an invitation letter and a consent form. They could choose between paper-pencil format and digital format when answering the survey. After two reminders, a total of 495 people had completed the survey. Of these, 155 were excluded for not meeting the inclusion criteria (use of HA/CI) (Figure 1).

The Swedish Association of Hard of Hearing People (HRF) is the largest organization for people with HL in Sweden, with 21,000 members. In the second round of data collection, HRF members (with a valid e-mail address, aged 18–67 years) received an invitation to participate in the study. In total, approximately 1197 people received and opened the e-mail (Figure 1). Two reminders to participate were sent. Interested members contacted the first author and, after providing informed consent, they received an e-mail with a link to the internet-based survey. A total of 258 people completed the survey. Of these, 22 were excluded as they did not meet the inclusion criteria.

The final sample consisted of 576 people.

### Analysis

The analysis was carried out using the IBM Statistical Package for the Social Sciences (SPSS) version 27.

#### Participant characteristics

Descriptive statistics (frequencies) were used to describe participant characteristics, the use of HAs/CIs, and WL information such as occupation, employment level and change of occupation or workplace.

To describe WL information, the participant's employment level (in percent of full-time) was categorized as full-time (100%) or part-time (<100%). Furthermore, because current occupation was an open-ended response option, the participants’ answers were classified according to so-called SSYK codes (Swedish Standard Classification of Occupations). This is based on ISCO-08 (International Standard Classification of Occupations), which is widely used in the EU for reporting statistics on occupations. The SSYK has a hierarchical structure with four different levels.^35^ The higher the level, the more detailed the description of the occupation. Each participant's occupation was coded at all levels. Participants who did not describe their occupations in sufficient detail could not be classified and were treated as internal missing (n = 65) in the analysis. In this analysis, there were 429 participants in the “in work” group and 82 participants in the “HL-related sick leave” group.

#### SOC

To analyse SOC in relation to working conditions, the indices and questions were categorized into the three areas: *comprehensibility*, *manageability,* and *meaningfulness* (Table 1). In total, 10 indices were used to measure SOC. Nine of these were part of an already-existing questionnaire and were therefore handled according to the developers’ instructions. As described above, one index of work accommodations (from the *manageability* area) was created by the authors, with questions about accommodations related to HL. Cronbach's alpha was used to analyse the internal consistency of this index and gave a satisfactory score (0.86).

**Table 1.** Comparison between the different dimensions of SOC for the “in work” group and the “HL-related sick leave” group.

Dimensions and variables in SOC	“In work”		“HL-related sick leave”		
	n	mean rank	n	mean rank	Z	ES (*r*)
**Comprehensibility**						
Process of change (index WEMS)	421	259	75	191	−3.772*	0.169
**Manageability**						
Autonomy (index WEMS)	485	299	86	215	−4.341*	0.182
Skill development (index LFS)	466	290	84	198	−4.884*	0.2083
Time experience (index WEMS)	481	294	86	228	−3.429*	0.1440
Self-efficacy (index)	489	300	86	222	−4.000*	0.1668
Work Accommodations (own index)	392	234	81	254	−1.221*ns*	
ALDs facilitative	262	162	56	147	−1.128*ns*	
Rest and recovery	489	301	85	213	−5.367*	0.2234
Balance between WL and leisure time	490	304	86	198	−6.539*	0.2726
Energy-demanding tasks outside of work	488	284	86	309	−1.361*ns*	
**Meaningfulness**						
Supportive working conditions (index WEMS)	480	294	86	226	−3.543*	0.1489
Internal work experience (index WEMS)	484	301	86	197	−5.398*	0.2261
Salary (index LFS)	476	289	83	230	−3.037*	0.1285
Leadership (index LFS)	451	277	82	211	−3.565*	0.1544

* = *p* < .05

Statistical significance was analysed in two ways, depending on the level of measurement. Group differences concerning response options on a nominal level were investigated with chi-square test. The Mann-Whitney U test was used to investigate group differences in ordinal response options.

The statistical significance level for all the analyses was set to 5% (*p* < .05). Furthermore, the effect size was computed by calculating the Rosenthal correlation coefficient (*r)* for the Mann-Whitney U test, and the ϕ correlation coefficient for the chi-square test.^36,37^ The correlation coefficient was interpreted according to Bartz, 1999^38^; 0.00 < .02 (very low), 0.2 < 0.4 (low), 0.4 < 0.6 (moderate), 0.6 < 0.8 (strong), 0.8 < 1.0 (very strong).

## Results

### Participant characteristics

A total of 576 people participated in the study. There were significantly more women than men (59.9% and 40.1% respectively), both overall and in the “in work” and “HL-related sick leave” groups respectively (Table 2). There were no significant differences between the two groups in terms of age, educational level, or economic burden (Table 2).

**Table 2.** Description of the sample.

	“In work” (n = 490)		“HL-related sick leave” (n = 86)					
	*n*	*%*	*n*	*%*	*x²*	*ES* ϕ	*Z*	*ES r*
Gender* (n = 573)					9.389	0.1277	—	—
Women	280	57.3	63	75.0				
Men	209	42.7	21	25.0				
**Age groups** *ns* (n = 574)							−1.131	
18–29 years	25	5.1	6	7.1				
30–54 years	178	36.3	34	40.5				
55–67 years	287	58.8	44	52.4				
**Education level (highest)** *ns* (n = 574)							−1.119	
Elementary school	33	6.8	4	4.7				
2-year high school	95	19.5	15	17.4				
3–4 year high school	93	19.1	14	16.3				
Folk high school	15	3.1	3	3.5				
University <3 years	71	14.5	14	16.3				
University ≥3 years	181	37.1	36	41.9				
Occupation (SSYK)* (n = 521)					5.448	0.1022	—	—
Education	53	12.1	18	21.7				
Personal care workers	49	11.2	9	10.8				
Health care	42	9.6	7	8.4				
Other	294	67.1	49	59.7				
Employment rate* (n = 575)					12.247	0.1458	—	—
Full time (100%)	309	63.1	37	43.5				
Part time	181	36.9	49	57.6				
**Change in work**								
Change of occupation or workplace due to hearing-related problems (yes)* (n = 533)	69	15.0	24	32.9	13.978	0.1619	–	—
Change of occupation affected their possibility to stay active in WL (yes)* (n = 259)	48	22.5	13	28.3	—	—	−3.191	0.1983
**Economic burden**								
Difficulties handling regular expenses (yes) (n = 574)	35	7.2	10	11.6	2.009			
Difficulty dealing with an unexpected expenses (yes) (n = 571)	59	12.2	14	16.3	1.109			
**Hearing Aids (HAs)** *ns* (n = 575)					3.214			
Bilateral	369	75.5	57	66.3				
Unilateral	112	22.9	27	31.4				
**Cochlear Implants (CIs)** * (n = 567)					10.986	0.1392	—	—
Bilateral	6	1.2	2	2.4				
Unilateral	22	4.5	11	13.4				
**Usage of HA/CI** *ns* (n = 565)							−.707	
Daily basis	383	79.6	67	79.7				
Occasionally	97	20.1	16	19.0				
**HA/CI positive impact on QoL** *ns* (n = 565)							−1.759	
Yes	415	85.9	63	76.8				
Sometimes	59	12.2	15	18.3				
No	9	1.9	4	4.9				
Assistive listening devices (ALDs) * (n = 571)					5.098	0.094	—	—
Yes	207	42.7	48	55.8				
No	278	57.3	38	44.2				

In the total sample, almost 74.1% used bilateral HAs and 1.4% used bilateral CIs. Furthermore, 24.2% used HAs unilaterally and 5.8% unilateral CIs. Some participants used a combination of HAs and CIs, so the numbers dońt add up to 100.0%. The majority (79.6%) of the total sample used HAs/CIs daily. In the total sample, 84.6% of participants reported that HAs/CIs had a positive effect on their health-related QoL. There was no significant difference in frequency of HAs/CIs, daily use of HAs/CIs, and positive impact of HAs/CIs on QoL between the “in work” and “HL-related sick leave” groups.

ALDs were used by 44.6% (n = 257) of the entire sample. Fewer participants in the “in work” group used ALDs than in the “HL-related sick leave” group. This difference was significant (Table 2).

Most of the participants (13.6%, n = 71) worked in *occupations requiring advanced academic competence in education* followed by *personal care workers* (11.1%, n = 58), and *occupations requiring advanced academic competence in health care* (9.4%, n = 49). When the “in work” and “HL-related sick leave” groups were analysed separately, the same occupational commonality was found. However, there was a significant difference between the groups concerning the most common occupation (Table 2).

In the entire sample, 346 people (60.2%) worked full-time. A significantly higher proportion of those “in work” worked full-time (Table 2).

Approximately 17.4% of the entire sample had changed their occupation or workplace due to hearing-related problems. Of these, 23.6% reported that the change of occupation affected their possibility to stay active in WL in an entirely positive way. It was more common to have changed workplace and for the change to have had a positive effect on the ability to remain active in WL in the “HL-related sick leave” group than in the “in work” group. The differences between the “in work” and “HL-related sick leave” groups were significant for both variables (Table 2).

### Working conditions in relation to SOC

The participants’ overall SOC was analysed in relation to working condition. The results demonstrated significant differences in all three dimensions, with a higher SOC for the “in work” group (Table 1).

Regarding the area of *comprehensibility*, the analysis showed that the “in work” group had a significantly higher mean rank than the “HL-related sick-leave” group. As comprehensibility covers different aspects of the process of change in an organization, a higher mean rank implies that participants experienced a higher understanding and comprehensibility of the work situation during the change process. They also felt a higher sense of participation during the process of change (Table 1).

Within the area of *manageability,* there were significant differences between the two groups (Table 1), with the “in work” group showing a higher mean rank and consequently a greater ability to manage and cope with the work situation using adequate resources. There was a significant difference between the two groups in relation to the single question on the use of ALDs, with those on “HL-related sick leave” using them to a greater extent than the other group (Table 2). However, no other significant differences were found in relation to the index hearing-related accommodations as a facilitative factor.

Within the dimension of *meaningfulness*, the analysis showed that the “in work” group had a significantly higher mean rank on all variables. Thus, the results indicates that those “in work” considered it more worthwhile to commit to, and being involved in, the work situation compared to those in the “HL-related sick leave” group.

## Discussion

Previous research on the WL of employees with HL has mainly focused on risk factors and the negative consequences of HL, especially the quantitative studies, with their lesser focus on salutary factors for a sustainable work situation. However, salutary factors may not be symmetrical to risk factors, which is why it is important to understand which factors, apart from risky working conditions, influence how workers with HL experience salutary working conditions. The results of the current study demonstrated that the “in work” group experienced a more salutary working life, as defined by a stronger SOC, than the “HL-related sick leave” group.

### Participant characteristics

#### Sick leave

Overall, one in six participants had been on HL-related sick leave in the most recent year. It was more common for women to have been on sick leave than men. This is consistent with the statistics on sick-leave for the general population in Sweden since the mid-1980s, with the same gender pattern: that it is more common for women to be on sick leave.^39^ This has also been shown by previous research on employees with HL.^10,11,40^ However, previous studies of sick leave among people with HL have not specified the cause of sick leave,^9^ and its relation to HL is therefore unknown. Furthermore, studies have used Swedish registry data on sick leave caused by various otoaudiological diagnoses.^40^ However, the use of registry data as a source for cause of sick leave in relation to HL can be problematic because the available statistics are inconclusive. The reason for this is that it is common to be on sick leave for a health condition other than HL, even if HL is the primary cause, or a mediating cause of the condition (e.g., mental strain or fatigue originating from the effort of listening in non-accommodated work environments).

### Working conditions in relation to SOC

In the salutogenic approach, it is important to understand health-promoting factors, i.e., what actively supports health and well-being.^25^ In this study, several health-promoting factors or resources related to SOC were found. The analysis showed significant differences in all three areas: comprehensibility, manageability and meaningfulness, where the “work” group reported a higher SOC than the “HL-related sick leave” group. This result is in line with SOC comparisons between in-work/sick-leave groups in the general population. Hansen et al.^26^ compared resources for return to work between an actively working population and a population on sick leave. They found that the actively working population had a higher SOC than those on sick leave. Additionally, job strain and SOC, and the association between job strain and perceived stress and depressed mood, have been investigated among teachers in Sweden, with high strain and low SOC being associated with both perceived stress and depressive mood. In the current study, occupations within the education sector are associated with a higher incidence of HL-related sick leave, indicating a need for health promotion in this occupational area to reduce sick leave in different working populations. It has also been concluded that high SOC may act as a protective factor against work-related stress.^25^ In Sweden, work-related stress has been reported to be a contributing factor in sick leave periods.^13^ Consequently, a high SOC, which acts as a buffer/protector against stress, may decrease the risk of sick leave. Indeed, a healthy work environment has been associated with a higher SOC when investigating the work environment of nurses,^41^ and this association also applies to employees with HL, as the results demonstrate associations between SOC and a healthy work environment. For employees with HL, this indicates that a focus on health-promoting work environments that acknowledge both auditory and non-auditory consequences of HL, and are therefore described as “HL-friendly”, could result in better working conditions for employees with HL, thereby increasing their chances of being active in work.

All participants in the current study had HL. In previous studies, challenges in WL and problems related to working conditions have mostly focused on the auditory consequences of HL, such as the use of HAs or ALDs.^42,43^ These two studies show that both audiologists and employees with HL perceive that the main focus in audiological rehabilitation is on technical aids, and that the challenging non-auditory aspects of WL are given less attention. The use of SOC as a concept in relation to working conditions has, to our knowledge, not been considered in scientific studies in relation to this target group. The current study demonstrates that employees with HL follow the same pattern with regard to SOC and sick leave as the general working population. Hence, the results imply the necessity of adopting a multidimensional perspective that takes into account both auditory and non-auditory consequences of HL, when addressing salutary working conditions for people with HL. The analysis in this study shows that when working conditions are explored in relation to SOC, the auditory consequences are subordinated to non-auditory consequences. This means that other generic aspects such as support, autonomy, and time experiences, are more strongly related to SOC than purely auditory consequences. Indeed, it has previously been shown that people with HL experience the same non-auditory factors to be important for the work environment.^10^ However, although they experience the same salutary factors as the general population, it has been argued that there is a difference in magnitude regarding this, when employees with HL are compared to other employees.^10,11^ For example, employees with HL may experience a greater need for control over their work tasks and a greater need for social support than the general working population. Qualitative studies support the need for control and support, as employees with HL highlight the importance of both aspects of the work environment,^5^ but whether the magnitude of these needs is greater for employees with HL than for other employees remains to be studied.

#### Comprehensibility

Comprehensibility concerns whether the employee perceives the work situation as structured, consistent, and clear, all of which can be considered health-promoting factors in the work situation. Within the dimension of *comprehensibility*, employees’ experiences of organizational change were analysed using an index that assesses the process of change. The process of organizational change, i.e., how an employee understands and deals with organizational changes, has been found to be a meaningful factor in salutary working conditions for a general population.^29^ In Sweden, organizational changes in the workplace are common due to the way WL is organized^44^ and thus are relevant to measure in relation to SOC.

The process of organizational change can create a stressful situation for employees who feel unable to predict how their work will be affected.^45^ In addition, change processes in relation to HL in WL can be a challenging factor, because missing out on information during meetings, or reading incomplete meeting notes, can result in a fragmented picture of the situation, and thus reduce their comprehension of the subject at hand. Indeed, missing information at meetings has been described as problematic for people with HL, resulting in reduced participation.^5^ In the current study, the “in work” group experienced a more comprehensive understanding of the work situation during a process of change than the “HL-related sick leave” group. The results demonstrate that for processes of change at work to be associated with a high SOC, the employees need to have knowledge about the change and a sense of inclusion in the process. This is in line with previous studies of SOC in relation to processes of change in a general working population, which show that an open dialogue about why and how the changes are being made is important in terms of increasing the employees’ sense of inclusion and influence during the process, as well as their understanding of their future role.^46^ In addition, the results of the current study showed that the “in work” group experienced that their managers were able to inform them about the changes in an understandable way, and thus increased their comprehension of the importance of the changes to a greater extent than the “HL-related sick leave” group. Furthermore, it is also possible that the group “HL-related sick leave” missed valuable information during their sick leave which resulted in a reduced understanding of the process of change. Even so, as one common auditory consequence of HL is missing information,^6^ it is interesting that the ability to assimilate and use information about the process of change differed between the groups. Assimilating information can be managed with coping strategies, one example being a so-called “buddy system”, where the person with HL receives informal help from a colleague to get complete information.^5,47^ A plausible explanation for the findings could therefore be that the “in work” group makes greater use of such coping strategies, one of which could be a “buddy system”. Indeed, in the general working population, a higher level of coping resources has previously been found among those active in work than among those on sick leave.^26^ With useful coping strategies, the “in work” group's comprehension and understanding of the work situation may increase, and this, in turn, may make it easier for them to manage and cope with the work situation.

#### Manageability

Manageability refers to internal and external resources and whether and how the employee uses these resources to meet the work demands. The analysis shows that the “in work” group reported higher manageability on indices measuring *self-efficacy, autonomy, time experience,* and *skills development.* All of these factors could be considered health-promoting, suggesting that the experience of having more of these resources is useful in managing the work situation. Higher self-efficacy can be interpreted as greater confidence in their ability to cope with new situations and problems and challenges that arise, compared to those with HL-related sick leave. This finding agrees with the results of a study by Cabrera-Aguilar,^48^ who investigated self-efficacy as a mediator of resilience and work engagement in nurses. The author concluded that self-efficacy increased the nurses’ ability to cope with work demands and also improved their engagement. Consequently, higher self-efficacy coupled with a greater sense of control over one's work in terms of flexibility and competence (skills development) regarding how and when tasks should be performed (autonomy), as well as the ability to keep up with the work during normal working hours (time experience), could help employees with HL to cope with new situations that arise at work. As expected, a work situation with flexible working hours and tasks has been reported to be important for employees, as flexibility leads to less strain and a higher degree of participation in WL, both for employees with HL^5^ and for the general working population.^26^ In the current study, the “at work” group reported significantly more perceived rest and recovery and a sounder balance between *WL and leisure* time than the “HL-related sick leave” group, both of which can be considered health-promoting factors. This result is in line with a recent study investigating different health factors for a sustainable work situation.^21^ The study reported that the “in work” group experienced fewer problems with items associated with mental strain, such as rest and recovery, and balance between WL and leisure time, than the “HL-related sick leave” group. Furthermore, the findings are confirmed by a focus-group study of workplace health resources in the general population, analysed using the concept of SOC.^49^ Nilsson et al. found that the ability to disconnect from work in leisure time led to better recovery and improved SOC. It has been argued that people with HL have a double workload in communicatively demanding situations where they have to analyse, manage and act on information received.^11,12^ This double workload leaves less cognitive capacity available for other aspects such as problem solving and learning,^50^ and leads to higher perceived effort and strain than for people with normal hearing.^17,51^ Energy-demanding listening results in greater effort and strain, as well as higher levels of fatigue, leading to a considerable need for recovery after a day's work.^6,17,52,53^ The need for recovery implies that social activities must be reduced to enable the person with HL to recover from work and manage the next workday.^6,52,53^ Salutary workplace factors may therefore include a flexible work situation and adjustments to the workday, such as taking breaks after energy-demanding listening to recover more easily from the cognitively demanding situation at work. In the current study, factors such as sense of control over the work situation, to get more rest and recovery, and have a better work-life balance is associated with a higher SOC. These factors can function as a health resource to help workers with HL manage their workday, resulting in better working conditions.

In relation to the domestic burden, the two groups experienced no significant difference regarding energy-demanding tasks outside work, although the “HL-related sick leave” group was shown to engage in these kinds of activities more often. The “HL-related sick leave” group also had a higher percentage of participants in the 30–54 age group than the “in work” group. In the question about domestic burden, the expression “energy-demanding tasks outside work” could be interpreted in various ways by different participants. One hypothesis is that although participants with families and children living at home have more energy-demanding tasks outside of work, they may not consider family life to be an energy-demanding task, as they consider it to be simply a part of life. However, energy-demanding tasks, at work as well as at home, do affect the ability to recover, and thus have a negative impact on health in WL.

The results also showed that the use of ALDs was more common in the “HL-related sick leave” group than in the “in work” group. ALDs decrease the auditory consequences of the HL, which may also have an impact on the non-auditory consequences, but they should not be viewed as a solution to all the problems in the WL. In order to make WL more manageable for this target group, these basic interventions need to be combined with other interventions. This is also the conclusion of previous studies, which reported that ALDs alone are not sufficient for overcoming employees’ difficulties at work.^34,43^ Vocational rehabilitation for people with HL requires a contextual bio-psycho-social approach that focuses on the individual and his/her specific work environment. A more comprehensive rehabilitation approach emphasizing work-related issues for employees with HL in WL has previously been argued to be important.^5,10,21,54^

In Sweden, the hearing healthcare services are responsible for the rehabilitation of all people with HL, including adults who are active in WL. The main professional group providing hearing health care is audiologists. However, specific work-related interventions are not a part of the undergraduate training programme for audiologists in Sweden. It can therefore be assumed that audiologists may be uncertain about which work-related aspects to focus on when working with clients who are active in the workplace. Internationally, this has been investigated by Zuriekat et al.,^43^ who examined audiologists’ experiences of working with patients who were active in WL. The study reported that the reasons why clinicians focus mainly on technical aids included their own lack of knowledge about the resources available to patients, lack of education and specific training in relation to the working population, and lack of knowledge about organizational characteristics. Specifically, the audiologists were unsure about which rehabilitation discipline/s were responsible for providing work-related rehabilitation to employees with HL. This latter finding has also been presented by Granberg and Gustafsson in a scoping review on HL in WL.^20^

The analysis in the current study showed that items addressing auditory consequences of HL, i.e., using work accommodations and experiencing whether ALD:s was facilitating, were not significantly related to SOC. The use of ALD was more common in the group “HL-related sick leave”, but there were no differences between the groups concerning the index work accommodations and the question measuring facilitating experiences of ALD. The use of ALD and work accommodations do not seem to increase the opportunities to be in work, nor to be related to SOC. From the results, it can be assumed that the auditory consequences were subordinated to the non-auditory consequences when analysing the situation in terms of the concept of SOC. Hence, to provide support for a salutary working situation, the non-auditory consequences of HL in WL – aspects such as anxious of losing one's job, missing out on important information, social isolation and/or exhaustion – need to be addressed in hearing health care.

#### Meaningfulness

Meaningfulness includes the extent to which the employee finds it worthwhile to invest energy in engagement and commitment at work. The analysis showed that the “in work” group reported a higher sense of meaningfulness on all indices than the “HL-related sick leave” group. Meaningfulness concerned work experiences related to *support from managers and colleagues, internal work experience* and *salary,* all of which can be considered health-promoting factors. The “in work” group experienced more support from managers and colleagues than the “HL-related sick leave” group. This is consistent with previous studies where support and feedback about one's work has proven to be important for SOC,^49,55^ and salutary working conditions^56^ in a general population. Moreover, receiving support has been shown to be important for good working conditions for employees with HL.^5,57^

In previous research on WL for employees with HL, support in the form of flexible working hours or tasks has been reported.^57^ However, the focus has mostly been on support from managers and colleagues in relation to increase the audibility^5,16,47,57^ and thus mainly concerned the auditory consequences of HL. In the current study, the questions in the index on support from managers concern different aspects of support related to non-auditory consequences of HL, such as appreciation, conflict management, how managers distribute work tasks, and responsiveness to employees’ views. The results demonstrated that support from colleagues, such as feedback, acknowledgement, and advice regarding work tasks, is important for the SOC of employees with HL. This is in line with previous research on salutary working conditions were receiving feedback and support in relation to ones performance at work can increase the feeling of doing good and also challenge you to develop in different work tasks.^55^ In this study, support from both managers and colleagues was assessed in relation to the non-auditory consequences of HL, and the findings demonstrate the importance of such support, as it seems to increase the possibility for employees with HL to remain in WL, without periods of HL-related sick leave.

Furthermore, in the current study, the “in work” group seems to experience a higher *internal work experience.* This is in line with previous research investigating salutary environmental factors for nurses, which showed that variation in work tasks and positively challenging situations, job satisfaction, and going to work with a positive feeling were important for SOC.^55^ In addition, a high SOC was associated with high levels of both social support and work engagement among nurses,^24^ an association that is also demonstrated in the current study, for employees with HL.

Consequently, the results in the current study demonstrate that those who are “in-work” experience more salutary working conditions and better work-life balance, resulting in higher SOC. Thus, by focusing on factors related to health resources and capabilities, both individually and workplace related, the results provide a different perspective on what matters in relation to health and well-being at work than a sole focus on hearing loss and disability. Such a salutogenic work perspective can thus provide both employees with HL and their employers, as well as hearing health care and occupational health services, with a set of health-promoting factors that, when implemented in the workplace, can contribute to a healthy and sustainable WL for employees with HL.

## Conclusion

The current study explored the concept of SOC in relation to working conditions for employees with HL by comparing two groups: adults with HL “in work” and on “HL-related sick leave”. The results showed that higher levels of SOC were experienced by those in the “in work” group compared to the “HL-related sick leave” group for all three SOC dimensions; higher comprehensibility, higher manageability and higher meaningfulness. This indicates that the “in work” group to a higher extent perceives the work situation as structured, consistent, and clear, uses different resources to meet the work demands and feel a sense of meaningfulness in the work situation, as well as considered the work situation worthy of commitment and involvement than the “HL-related sick leave” group. Consequently, the result suggest that SOC is a valuable concept for studying working conditions for employees with HL. Living with HL involves auditory and non-auditory challenges and has consequences for everyday life. In relation to the working conditions analysed here with SOC, the auditory consequences were subordinated to the non-auditory consequences. Consequently, the results indicate that to increase the possibility of a salutary working situation, one needs to focus on the non-auditory consequences of HL such as recovery, autonomy and support. Therefore, both hearing health care providers and employers need to also address non-auditory consequences of HL in WL, such as social isolation, missing out on important information, exhaustion, and/or fear of losing one's job, in order to increase the chances of achieving salutary working conditions for employees with HL. If non-auditory aspects of the work situation are addressed, the employees’ comprehension and ability to manage the work situation and sense of meaningfulness increase, which may protect against work-related stress and reduce the risk of sick leave.

### Strengths and limitations

A strength of this study was the use of validated questions and/or indices previously used in research in the field of WL and health. Some questions regarding aspects of HL, HL-related sick leave, ALDs and work accommodations were developed by the authors. The purpose of this was to construct questions of specific relevance to the target group of the study. These self-developed questions have been used in a previous study.^21^ Furthermore, the aim of the study was to explore working conditions for employees with HL in relation to the theory/concept of SOC. Concerning work-related SOC, a Work-SOC questionnaire has been developed.^58^ This is a generic questionnaire designed to assess how the employee perceives his or her current job and work situation. However, as certain specific factors have been shown to be important for the target group in the study of WL,^5,20,21^ it seemed important to include questions addressing these factors in the questionnaire of the current study. Therefore, instead of using the general Work-SOC questionnaire, questions of specific relevance for the target group made up the questionnaire and were subsequently analyzed using the SOC theory. For the same reason, the entire WEMS questionnaire was not included in the analysis. Instead, the sub-scale from the LFS concerning leadership was used. Previous research has shown that support from managers is important for employees with HL.^5^ The sub-scale leadership from LFS has a greater focus on supportive management compared to the corresponding sub-scale in WEMS. These questions were therefore determined to have a higher relevance for the target group of the current study.

Another strength of this study is that the participants were recruited from all parts of Sweden. Approximately 250 participants were recruited from the HRF, which is the largest association for people with hearing impairment in Sweden. It is possible that the members of this association are not representative of the general population of employees with HL, due to their involvement and knowledge about HL and its consequences. However, approximately 400 participants in the current study were recruited from an audiological clinic, and they may be more representative of the general population of employees with HL. A separate analysis was carried out to identify differences between the two subpopulations (national and clinical). The results indicated small differences on a few questions, suggesting that differences between the subpopulations were not substantially related to the main area of focus: SOC. It was decided that the advantage of merging the two subpopulations to get a larger sample spread across Sweden outweighed the small differences between the two populations. Therefore, the two sub-populations were merged and analysed as a single national population. Its strength as a national population is that the participants come from all over the country, and the effects of any local or regional differences are minimized. Combining the two sub-populations (national and clinical) may therefore even out the differences regarding knowledge about HL and its consequences. The study used convenience sampling, which has been criticized for difficulties related to generalization, because the participants might not be typical of the population being studied.^59^ However, the combination of the national and clinical populations should increase the representativeness of the sample and thus the generalizability of the results. A potential limitation of this study relates to the measurement of effect size. This type of measure is somewhat problematic in connection with cross-sectional studies. However, effect size can reveal tendencies and thereby guide the interpretation of the results, and is therefore a valuable measure to include in an analysis of this kind.

## References

[1] WHO. World report on disability. Geneva: World Health Organization, 2011.

[2] Waddell G Burton K . Is work good for your health and well-being. 2006.

[3] The Global Goals. The 17 goals: The Global Goals; nd [cited 2023]. Available at: https://www.globalgoals.org/goals/.

[4] Emmett SD Francis HW . The socioeconomic impact of hearing loss in U.S. Adults. Otol Neurotol 2015; 36: 545–550. 25158616 10.1097/MAO.0000000000000562 PMC4466103

[5] Svinndal EV Jensen C Rise MB . Working life trajectories with hearing impairment. Disabil Rehabil 2020; 42: 190–200. 30298745 10.1080/09638288.2018.1495273

[6] Hua H Anderzen-Carlsson A Widen S , et al. Conceptions of working life among employees with mild-moderate aided hearing impairment: a phenomenographic study. Int J Audiol 2015; 54: 873–880. 26140299 10.3109/14992027.2015.1060640

[7] The Swedish Association of Hard of Hearing People. Hearing loss in figures 2017. Statistics on people with hearing loss and prescriptions of hearing aids from the Swedish Association of the Hard of Hearing (HRF), version 2.1 [Internet]. Stockholm: HRF, 2017. [cited 2025 Jan 29]. [Swedish]. Available at: https://hrf.se/app/uploads/2016/06/Hsk_i_siffror_nov2017_webb.pdf

[8] Montano J . Audiologic rehabilitation. In: Katz J Chasin M English KM , et al. (eds) Handbook of clinical audiology. 7th ed. Philadelphia: Wolter Kluwer, 2015, pp.849–860.

[9] Svinndal EV Solheim J Rise MB , et al. Hearing loss and work participation: a cross-sectional study in Norway. Int J Audiol 2018; 57: 646–656. 29703092 10.1080/14992027.2018.1464216

[10] Danermark B Gellerstedt LC . Psychosocial work environment, hearing impairment and health. Int J Audiol 2004; 43: 383–389. 15515637 10.1080/14992020400050049

[11] Coniavitis Gellerstedt L Danermark B . Hearing impairment, working life conditions, and gender. Scand J Disabil Res 2004; 6: 225–245.

[12] Gullacksen A-C . Hearing impaired in working life – a stress/control perspective [licentiate thesis]. Lund: Lund University, School of social work, 1993 [Swedish].

[13] The Swedish Social Insurance Agency. Mental health in today’s working life [Internet]. Sweden: The Swedish Social Insurance Agency, 2023; Short analyses; 2023:6. [cited 2025 Jan 29]. [Swedish]. Available at: https://www.forsakringskassan.se/download/18.21e4089719320208e5873/1731686616744/psykisk-ohalsa-i-dagens-arbetsliv-lagesrapport-2024.pdf.

[14] Jørgensen AY Aarhus L Engdahl B , et al. Hearing loss, sick leave, and disability pension: findings from the HUNT follow-up study. BMC Public Health 2022; 22: 1340. 35836216 10.1186/s12889-022-13760-2 PMC9281024

[15] Swedish Work Environment Authority. Organisational and social work environment. 2015.

[16] Shaw L Tetlaff B Jennings MB , et al. The standpoint of persons with hearing loss on work disparities and workplace accommodations. Work (Reading, Mass) 2013; 46: 193–204. 24004807 10.3233/WOR-131741

[17] Kramer SE Kapteyn TS Houtgast T . Occupational performance: comparing normally-hearing and hearing-impaired employees using the Amsterdam checklist for hearing and work. Int J Audiol 2006; 45: 503–512. 17005493 10.1080/14992020600754583

[18] Punch R . Employment and adults who are deaf or hard of hearing: current Status and experiences of barriers, accommodations, and stress in the workplace. Am Ann Deaf 2016; 161: 384–397. 27477043 10.1353/aad.2016.0028

[19] Jennings MB Shaw L . Impact of hearing loss in the workplace: raising questions about partnerships with professionals. Work (Reading, Mass) 2008; 30: 289–295. 18525152

[20] Granberg S Gustafsson J . Key findings about hearing loss in the working-life: a scoping review from a well-being perspective. Int J Audiol 2021; 60: 60–70. 10.1080/14992027.2021.1881628 33630697

[21] Granberg S Widen S Gustafsson J . How to remain in working life with hearing loss - health factors for a sustainable work situation. Work (Reading, Mass) 2024; 79: 1391–1406. 38875067 10.3233/WOR-230377 PMC11613010

[22] Jenny GJ Bauer GF Vogt K , et al. The application of salutogenesis to work. In: Mittelmark MB Sagy S Eriksson M , et al. (eds) The handbook of salutogenesis. Cham (CH): Springer, 2017, pp. 197–210. 28590629

[23] Antonovsky A . Unraveling the mystery of health: how people manage stress and stay well. 1 ed. San Fransisco: Jossey-Bass, 1987.

[24] Malagon-Aguilera MC Suñer-Soler R Bonmatí-Tomas A , et al. Relationship between sense of coherence, health and work engagement among nurses. J Nurs Manag 2019; 27: 1620–1630. 31444895 10.1111/jonm.12848

[25] Ramberg J Låftman SB Nilbrink J , et al. Job strain and sense of coherence: associations with stress-related outcomes among teachers. Scand J Public Health 2022; 50: 565–574. 33977811 10.1177/14034948211011812 PMC9203657

[26] Hansen A Edlund C Bränholm IB . Significant resources needed for return to work after sick leave. Work (Reading, Mass) 2005; 25: 231–240. 16179772

[27] Public Health Agency of Sweden. Public Health Reporting: Public Health Agency of Sweden; nd. Available at: https://www.folkhalsomyndigheten.se/the-public-health-agency-of-sweden/public-health-reporting/.

[28] Downward P Rasciute S Kumar H . Health, subjective financial situation and well-being: a longitudinal observational study. Health Qual Life Outcomes 2020; 18: 203. 32590985 10.1186/s12955-020-01456-3 PMC7318449

[29] Nilsson P Bringsen A Andersson HI , et al. Development and quality analysis of the work experience measurement scale (WEMS). Work (Reading, Mass) 2010; 35: 153–161. 20164610 10.3233/WOR-2010-0967

[30] Ericsson U . On organizing the regenerative work: conversations about role, process and interactive meaning-making [PhD dissertation on the Internet]. Stockholm: Royal Institute of Technology, KTH, Industrial Economics and Management, 2010. [cited 2025 Jan 29]. [Swedish]. Available at: https://www.diva-portal.org/smash/get/diva2:322103/FULLTEXT01.pdf

[31] Statistics Sweden. Labour Force Surveys (LFS): Statistics Sweden; nd. Available from: https://www.scb.se/en/finding-statistics/statistics-by-subject-area/labour-market/labour-force-surveys/labour-force-surveys-lfs/.

[32] Lönnfjord V Hagquist C . The psychometric properties of the Swedish version of the general self-efficacy scale: a rasch analysis based on adolescent data. Curr Psychol 2018; 37: 703–715. 30416321 10.1007/s12144-016-9551-y PMC6208847

[33] Trap R Rejkjær L Hansen EH . Empirical relations between sense of coherence and self-efficacy, national danish survey. Health Promot Int 2016; 31: 635–643. 26069296 10.1093/heapro/dav052

[34] Bjarnason S . The job is communication: on the use of assistive listening devices at work for people with hearing loss [Licentiate thesis on the Internet]. Örebro: Örebro University, Studies from The Swedish Institute for Disability Research, 2011. [cited 2025 Jan 29]. [Swedish]. Available from: https://urn.kb.se/resolve?urn=urn:nbn:se:oru:diva-20593

[35] Statistics Sweden. Introduction SSYK 2012: Statistics Sweden; nd. Available at: https://www.scb.se/contentassets/0c0089cc085a45d49c1dc83923ad933a/in-english-ssyk-2012.pdf.

[36] Fritz CO Morris PE Richler JJ . Effect size estimates: current use, calculations, and interpretation. J Exp Psychol Gen 2012; 141: 2–18. 21823805 10.1037/a0024338

[37] Tomczak M Tomczak E . The need to report effect size estimates revisited. An overview of some recommended measures of effect size. Trends Sport Sci 2014; 1: 19–25.

[38] Bartz AE . Basic statistical concepts. 4 ed. Upper Saddle River, NJ: Merrill, 1999.

[39] Försäkringskassan. Social Insurance in Figures 2023. Försäkringskassan; 2023 2024.

[40] Friberg E Rosenhall U Alexanderson K . Sickness absence due to otoaudiological diagnoses; a descriptive nationwide study. BMC Public Health 2013; 13: 635. 23835212 10.1186/1471-2458-13-635 PMC3733665

[41] Ogata Y Sato K Sasaki M , et al. Association between nursing practice environment and sense of coherence among staff nurses: a cross-sectional study in Japan. J Nurs Manag 2022; 30: 3149–3159. 35781366 10.1111/jonm.13733

[42] Zuriekat M Alqudah S Semeraro H , et al. The audiological rehabilitation of workers with hearing loss in the UK: a qualitative study of workers’ perspectives. Disabil Rehabil 2024; 46: 3946–3960. 37800442 10.1080/09638288.2023.2261375

[43] Zuriekat M Semeraro H Watson V , et al. Hearing healthcare for workers with hearing loss: audiologists’ experiences and views. Disabil Rehabil 2022; 44: 7861–7871. 34817312 10.1080/09638288.2021.2001053

[44] Hultberg A Ahlborg G Winroth J , et al. Health in the workplace – a compilation of knowledge and methods. Örebro: Institute for Stress Medicine, 2018. Report No.: ISM-report 21 [Swedish].

[45] Dellve L Eriksson A . Sustainable leadership - in everyday life and change. Borås: The university of Borås, 2016 [Swedish].

[46] Tvedt SD Saksvik PØ Nytrø KJW . Does change process healthiness reduce the negative effects of organizational change on the psychosocial work environment? Stress 2009; 23: 80–98.

[47] Jennings MB Southall K Gagné JP . Social identity management strategies used by workers with acquired hearing loss. Work (Reading, Mass) 2013; 46: 169–180. 24177389 10.3233/WOR-131760

[48] Cabrera-Aguilar E Zevallos-Francia M Morales-García M , et al. Resilience and stress as predictors of work engagement: the mediating role of self-efficacy in nurses. Front Psychiatry 2023; 14: 1202048. 37649562 10.3389/fpsyt.2023.1202048 PMC10464840

[49] Nilsson P Andersson IH Ejlertsson G , et al. Workplace health resources based on sence of coherence theory. Int J Workplace Health Manag 2012; 5: 156–167.

[50] Baddeley A . Working memory: theories, models, and controversies. Annu Rev Psychol 2012; 63: 1–29. 21961947 10.1146/annurev-psych-120710-100422

[51] Pichora-Fuller MK Kramer SE Eckert MA , et al. Hearing impairment and cognitive energy: the framework for understanding effortful listening (FUEL). Ear Hear 2016; 37: 5s–27s. 27355771 10.1097/AUD.0000000000000312

[52] Davis H Schlundt D Bonnet K , et al. Understanding listening-related fatigue: perspectives of adults with hearing loss. Int J Audiol 2020: 1–11. 10.1080/14992027.2020.1834631 33106063

[53] Holman JA Drummond A Hughes SE , et al. Hearing impairment and daily-life fatigue: a qualitative study. Int J Audiol 2019; 58: 408–416. 31032678 10.1080/14992027.2019.1597284 PMC6567543

[54] Kramer SE . Hearing impairment, work, and vocational enablement. Int J Audiol 2008; 47: S124–S130. 10.1080/14992020802310887 19012121

[55] Nunstedt H Eriksson M Obeid A , et al. Salutary factors and hospital work environments: a qualitative descriptive study of nurses in Sweden. BMC Nurs 2020; 19: 125. 33342433 10.1186/s12912-020-00521-y PMC7751112

[56] Aronsson G Lindh T . Long-term health workers’ working conditions. A population study [Internet]. Stockholm: National Institute for Working Life, 2004; Work and Health; 2004:10. [cited 2025 Jan 29]. [Swedish]. Available at: https://gupea.ub.gu.se/bitstream/handle/2077/4332/ah2004_10.pdf?sequence=1&isAllowed=y

[57] Svinndal EV Jensen C Rise MB . Employees with hearing impairment. A qualitative study exploring managers’ experiences. Disabil Rehabil 2020; 42: 1855–1862. 10.1080/09638288.2018.1541101 30669885

[58] Vogt K Jenny GJ Bauer GF . Comprehensibility, manageability and meaningfulness at work: construct validity of a scale measuring work-related sense of coherence. SA J Ind Psychol 2013; 39: e1–e8.

[59] Polit F Beck CT . Nursing research: generating and assessing evidence for nursing practice. 11 ed. Philadelphia: Wolters Kluwer, 2021.

